# Narrow-Band Imaging Improves Detection of Colorectal Peritoneal Metastases: A Clinical Study Comparing Advanced Imaging Techniques

**DOI:** 10.1245/s10434-018-7005-5

**Published:** 2018-11-12

**Authors:** Nina Roelie Sluiter, Stijn Lucas Vlek, Arthur Randolph Wijsmuller, Henk Thijs Brandsma, Henrica Cornelia Wilhelmina de Vet, Nicole Cornelia Theodora van Grieken, Geert Kazemier, Jurriaan Benjamin Tuynman

**Affiliations:** 1 Department of Surgery, Cancer Center Amsterdam, Amsterdam UMC, Vrije Universiteit Amsterdam, Amsterdam, The Netherlands; 2 Department of Epidemiology and Biostatistics, Amsterdam UMC, Vrije Universiteit Amsterdam, Amsterdam, The Netherlands; 3 Department of Pathology, Amsterdam UMC, Vrije Universiteit Amsterdam, Amsterdam, The Netherlands

## Abstract

**Background:**

Colorectal peritoneal metastases (PM) are often diagnosed in an advanced disease stage. Cytoreduction and hyperthermic intraperitoneal chemotherapy (HIPEC) improve survival of patients with colorectal PM, although most benefit is seen in patients with limited peritoneal disease. Advanced imaging techniques might improve the detection of PM, potentially leading to earlier diagnosis and improved cytoreduction. This prospective clinical trial compared three advanced techniques with conventional white-light imaging for the detection of colorectal PM: narrow-band imaging (NBI), near-infrared indocyanine green fluorescent imaging (NIR-ICG), and spray-dye chromoendoscopy (SDCE).

**Methods:**

Patients with colorectal PM were prospectively included. Prior to cytoreduction and HIPEC, all abdominal regions were inspected with white-light imaging, NBI, NIR-ICG, and SDCE during exploratory laparoscopy. Primary endpoints were sensitivity and specificity for the detection of PM, using pathological examination of biopsied lesions as the reference standard. The safety of all techniques was assessed.

**Results:**

Between May 2016 and March 2018, four different techniques were analyzed in 28 patients, resulting in 169 biopsies. Sensitivity for the detection of PM significantly increased from 80.0% with white light to 96.0% with NBI (*p* = 0.008), without loss of specificity (74.8% vs. 73.1%, respectively, *p* = 0.804). The use of NIR-ICG and SDCE was discontinued after 10 patients had undergone treatment because the lesions were not fluorescent using NIR-ICG, and because SDCE did not visualize the whole peritoneum. No adverse events relating to the imaging techniques occurred.

**Conclusion:**

NBI substantially increased the detection of PM. This method is safe and could improve the detection of metastatic lesions and help optimize cytoreduction in patients with colorectal PM.

**Electronic supplementary material:**

The online version of this article (10.1245/s10434-018-7005-5) contains supplementary material, which is available to authorized users.

Peritoneal metastases (PM) are diagnosed in 10–25% of colorectal cancer (CRC) patients^1^–^3^ and severely jeopardize survival. Patients with colorectal PM have a median overall survival of 4 months without treatment^4^ and 12–16 months after treatment with systemic chemotherapy.^5^^,^^6^ Currently, the only potentially curative option for patients with colorectal PM consists of cytoreductive surgery (CRS) and hyperthermic intraperitoneal chemotherapy (HIPEC). In carefully selected patients with limited peritoneal disease, a median overall survival of 45 months can be reached, resembling the survival rates of stage 3 CRC patients.^5^^,^^7^^,^^8^

Treatment with cytoreduction and HIPEC is associated with relatively high morbidity and mortality rates of 16–64% and 5%, respectively^9^–^11^ warranting careful selection of potential HIPEC candidates. Consequently, much of the current literature focuses on the identification of prognostic factors. Two major prognosticators associated with poor oncologic outcome are a high intraperitoneal tumor load and, inherently, an incomplete cytoreduction (R2).^1^^,^^12^^,^^13^ Accordingly, an earlier diagnosis of PM is key to further improving prognosis, warranting enhanced detection of PM during primary tumor resection. Second, it is crucial to achieve a complete cytoreduction to improve oncologic outcomes. Nevertheless, visualization of PM and quantification of the peritoneal tumor burden are challenging. The value of current preoperative imaging by positron emission tomography/computed tomography (PET/CT) is limited by its low sensitivity for detection of PM (72%),^14^ which decreases further to 11% for nodules smaller than 5 mm.^15^ Accordingly, intraperitoneal tumor detection and a complete cytoreduction rely on intraoperative staging based on visual detection of tumor nodules and palpation of the abdominal surface.

Advanced imaging techniques are increasingly used for visualization of several cancer types, in addition to white-light imaging, the conventional imaging technique, and could improve the detection of PM. Promising methods include narrow-band imaging (NBI), near-infrared imaging with indocyanine green (NIR-ICG), and spray-dye chromoendoscopy (SDCE) with indigo carmine blue. NBI consists of 415 and 540 nm wavelengths, and highlights microvascular architecture,^16^^,^^17^ thereby accentuating deviating patterns and demarking peritoneal nodules.^18^^,^^19^ Near-infrared imaging depends on intravenously administered indocyanine green (ICG) that accumulates in tumor tissue, resulting in fluorescence using near-infrared light.^20^–^22^ A third method is SDCE with the indigo carmine blue dye that accentuates the malignant architecture of tumor lesions and is mainly described for its use during gastroendoscopy and colonoscopy.^23^^,^^24^

The modalities NBI, NIR-ICG, and SDCE have been shown to improve the detection of lesions in different medical fields of specialty. However, to date, NBI and SDCE have never been studied for the the detection of colorectal PM, and studies on NIR-ICG report contradictory results. Therefore, the present study aims to investigate the feasibility and safety of NBI, NIR-ICG, and SDCE for the detection of colorectal PM.

## Methods

### Surgery and Imaging Modalities

Patients with colorectal PM scheduled for CRS and HIPEC were prospectively enrolled in this clinical feasibility study. All included patients underwent a diagnostic laparoscopy prior to CRS and HIPEC, during which NBI, NIR-ICG, and SDCE were compared with white-light imaging. Details on study design, patients, and surgical procedures are provided in the electronic supplementary methods.

Per imaging modality, two predefined scoring systems were used: (1) lesions were scored as benign or malignant; and (2) lesions were scored using a visual analog scale (VAS) ranging from 1 (certainly benign) to 10 (certainly malignant). Benign lesions were scored as VAS 1–4, dubiously malignant lesions were scored as VAS 5–6, and malignant lesions were scored as VAS 7–10. The VAS indicated whether surgeons were more likely to classify a lesion as benign (low VAS) or malignant (high VAS). Guidelines for the assessment of lesions are provided per imaging modality in the electronic supplementary methods. Biopsies of all potentially malignant lesions and negative control biopsies within 2 cm proximity of these lesions were taken. Two independent gastrointestinal surgeons reviewed the photographs of all lesions in order to assess interrater variability, and a pathologist inspected all biopsies in a blinded fashion.

### Statistics

According to the sample size calculation (electronic supplementary methods), a sample size of 25–30 patients was required. Primary (sensitivity, specificity) and secondary outcomes (positive [PPV] and negative predictive values [NPV], positive [LR +] and negative likelihood ratios [LR −]) were calculated for each imaging modality, using pathological examination of the biopsies as the golden standard. Differences in sensitivity and specificity between the advanced imaging techniques and white-light imaging were compared using McNemar’s test, and differences in VAS for certainty of malignancy or benignancy were calculated using the Wilcoxon signed rank test. Statistical significance was assumed at a *p* value of < 0.05 for two-sided testing. Interrater variability was assessed using Cohen’s weighted kappa value (*K*_w_) with 95% confidence intervals (CIs). Statistical analyses were performed using SPSS version 22 (IBM Corporation, Armonk, NY, USA).

## Results

### Patients

Between May 2016 and March 2018, 40 patients were included in our study. Twelve patients were excluded because no PM were detected intraoperatively (*n* = 5) or because pathological examination showed the lesions to be of non-colorectal (*n* = 4) or low-grade appendiceal mucinous neoplasm [LAMN; *n* = 3) origin, leaving 28 eligible patients. Baseline characteristics of all patients are depicted in Table 1. Eight patients were considered inoperable based on the laparoscopic findings and hence did not undergo a CRS and HIPEC procedure. None of the patients experienced adverse events related to any of the techniques used, and none of the laparoscopic procedures were abandoned because of the presence of adhesions.

**Table 1:** Baseline characteristics of all patients

Characteristic	*N*/mean (SD)
General characteristics	
All	28
Female sex	13
Age, years	
Mean	64.3 (10.3)
Primary tumor characteristics	
Location	
Appendix	2
Colon	24
Rectum	2
Tumor differentiation	
Good/moderate	16
Poor	1
Signet cell	3
Goblet cell	1
Mucinous type	6
Synchronous PM	14
Stage	
2	6
3	9
4	13
Prior treatment	
Previous adjuvant chemotherapy	9
Prior surgical score	
0	23
1	3
2	2
HIPEC characteristics	
Operative procedure	
CRS and HIPEC	20
Only diagnostic laparoscopy	8
Reason for exclusion from CRS and HIPEC	
PCI too high	5
Irresectable primary	1
Liver metastases	1
Para-aortic lymph nodes	1
PCI	
Mean	13 (9)
Resection score	
R1	20
R2	8

*CRS* cytoreductive surgery, *HIPEC* hyperthermic intraperitoneal chemotherapy, *PCI* Peritoneal Cancer Index, *PM* peritoneal metastases, *SD* standard deviation

White-light imaging and NBI were studied in all included patients. ICG (0.25 mg/kg bodyweight) was administered 3 h (*n* = 4) or 12 h before surgery (*n* = 3). A double dose of ICG (0.5 mg/kg bodyweight) was administered 3 h before surgery in the last three patients; however, its use was discontinued after 10 patients had undergone treatment because neither of the used doses or time intervals resulted in fluorescent PM. SDCE was also discontinued after 10 patients had undergone treatment as application of the dye onto the whole peritoneum was time-consuming, and was impractical for application onto the whole abdominal cavity and intraperitoneal organs, impairing visualization of intra-abdominal organs.

### Scoring as Benign or Malignant: Primary Outcomes (Scoring System 1)

**Fig. 1:**
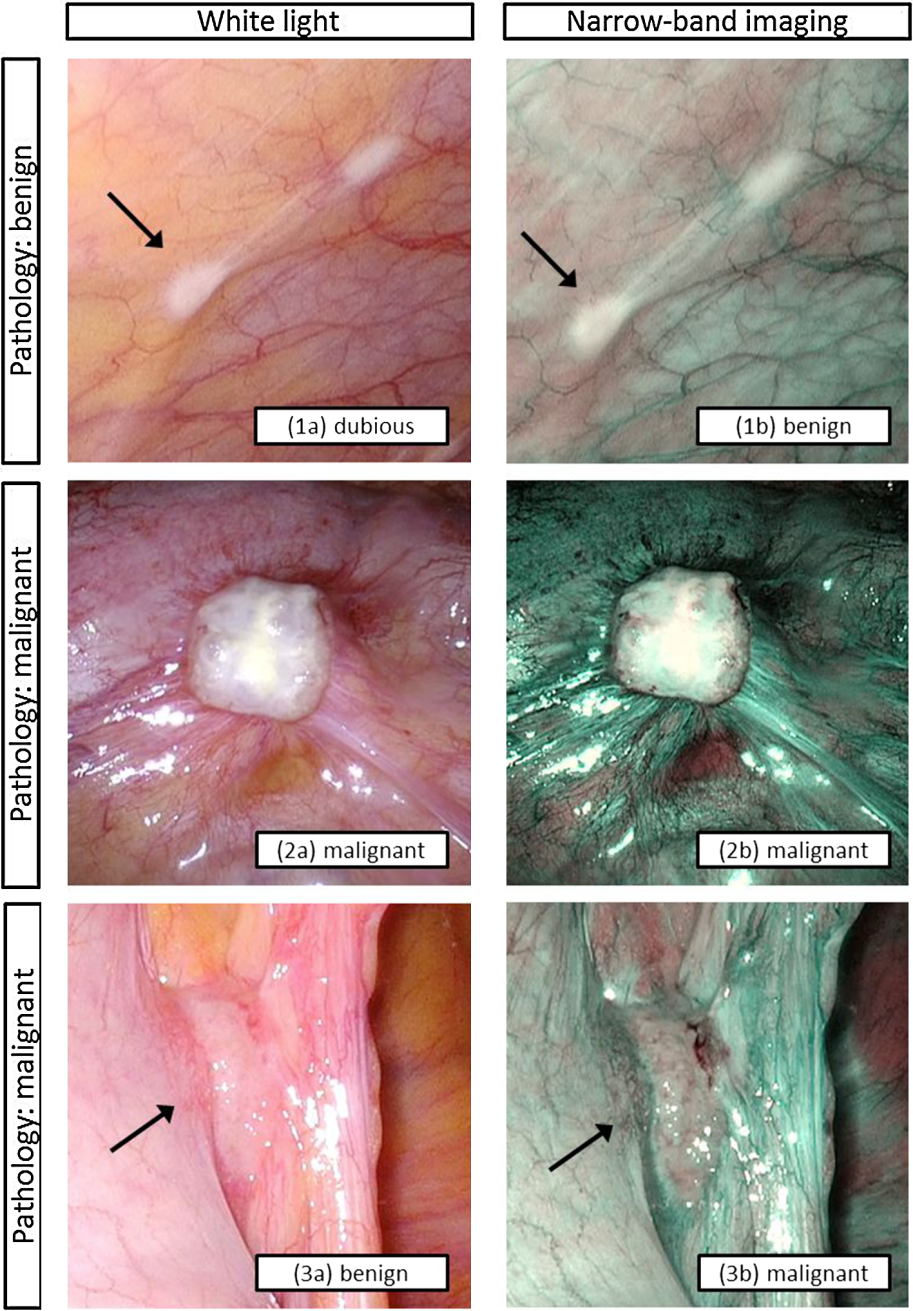
Examples of a benign lesion that was scored as (1a) dubious with white light, and (1b) benign with NBI (no vascular abnormalities); (2a, b) a malignant lesion that was scored as malignant with both white light and NBI (rich vascularization and brown spots); and (3a, b) a malignant lesion that was missed with white light and scored as malignant with NBI (brown spots). The arrows indicate the assessed lesions. *NBI* narrow-band imaging

A total of 169 biopsies were taken, of which 92 lesions were suspected for malignancy and 77 were control lesions. A mean of 6.0 biopsies (standard deviation [SD] 1.8) were taken per patient. Pathological examination revealed 50 malignant and 119 benign lesions. The mean size of all lesions was 6.7 mm (SD 6.6): 5.1 mm (SD 3.7) of pathologically confirmed benign lesions and 7.3 mm (SD 10.7) of pathologically confirmed malignant lesions. Blinded assessment of lesions by two independent surgeons, scoring lesions as either benign or malignant, demonstrated good interobserver agreement for white light (*K*_w_ = 0.62, 95% CI 0.41–0.83), and an excellent interobserver agreement for NBI (*K*_w_ = 0.78, 95% CI 0.54–1.02) and SDCE (*K*_w_ = 0.85, 95% CI 0.57–1.13).^25^ Figure 1 represents examples of benign and malignant lesions as depicted by the imaging modalities.

NBI significantly improved sensitivity from 80.0% with white-light imaging to 96.0% (*p* = 0.008, χ^2^ 8.0, 1 degree of freedom [*df*]), while specificity between the two techniques was comparable (white light 74.8% vs. NBI 73.1%, *p* = 0.804, χ^2^ 0.25, 1 *df*). None of the other imaging modalities significantly increased sensitivity. Combining white-light imaging with NBI did not increase sensitivity compared with NBI alone, but did result in a loss of specificity (67.2%, *p* = 0.004, χ^2^ 8.0, 1 *df*) compared with white-light imaging alone. Table 2 displays the primary outcomes for the imaging techniques. Using NBI, eight additional lesions were detected that were missed with white light and appeared malignant on pathological examination. No additional malignant lesions were detected with white light compared with NBI. The mean size of the malignant lesions missed with white light was 5.7 mm (SD 3.0) and the mean size of the lesions that were missed with white light but were detected with NBI was 5.4 mm (SD 3.0).

**Table 2:**
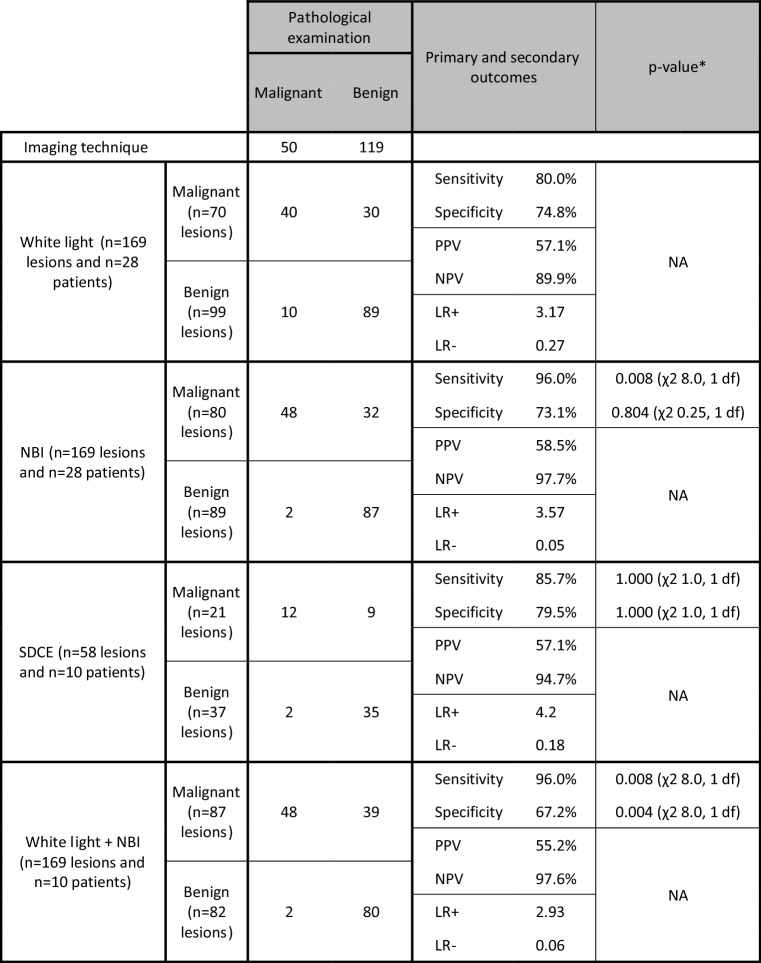
Primary and secondary outcomes

*LR* − negative likelihood ratio, *NBI* narrow-band imaging, *NPV* negative predictive value, *NA* not applicable, *LR* + positive likelihood ratio, *PPV* positive predictive value, *SDCE* spray-dye chromoendoscopy, *df* degree of freedom

^*^Two-sided McNemar’s test

### Scoring According to a Visual Analog Scale (Scoring System 2)

Table 3 represents the VAS assigned to all lesions. Blinded assessment of lesions, scoring these according to the VAS, demonstrated a fair to good interobserver agreement for both white light (*K*_w_ = 0.59, 95% CI 0.45–0.72) and NBI (*K*_w_ = 0.69, 95% CI 0.55–0.83). The VAS assigned to pathologically confirmed malignant lesions was lower when ranked with white light (7.4, SD 1.6) than when ranked with NBI (8.4, SD 1.9; *p* < 0.001) [*Z* = − 4.195, *df* not applicable]. The mean VAS assigned to pathologically confirmed benign lesions was slightly lower when ranked with white light (2.7, SD 2.5) than when ranked with NBI (3.2, SD 2.5; *p* = 0.004) [*Z* = − 2.784, *df* not applicable].

**Table 3:** Scoring of all lesions according to VAS scores for white light and NBI

	Pathology: benign	Pathology: malignant
White light VAS [mean (SD)]	2.7 (2.5)	7.4 (1.6)
NBI VAS [mean (SD)]	3.2 (3.2)	8.4 (1.9)
*p* value^a^	0.004 (*Z* = − 2.784, *df* NA)	< 0.001 (*Z* = − 4.195, *df* NA)

The VAS indicates whether a pathologically confirmed benign or malignant lesion is likely to be scored as benign (low VAS) or malignant (high VAS)

*NBI* narrow-band imaging, *NA* not applicable, *SD* standard deviation, *VAS* visual analog scale, *df* degree of freedom

^a^Wilcoxon signed rank test

Considering the three predefined categories according to the VAS (benign, dubious, malignant), 93 lesions (55.0%) were scored as benign, 19 (11.3%) were scored as dubious, and 57 (33.7%) were scored as malignant using white-light imaging (Table 4). Using NBI, 88 lesions (52.1%) were scored as benign, 2 (1.2%) were scored as dubious, and 79 (46.7%) were scored as malignant. Five of 93 lesions (5.4%) and 2 of 88 lesions (2.3%) scored as benign with white light or NBI, respectively, appeared malignant on pathological examination. Seven of 19 lesions (36.8%) scored as dubious using white light were found to be malignant at pathological assessment. All these seven lesions were classified as malignant using NBI. Thirty-eight of 57 lesions (66.7%) and 48 of 79 lesions (60.8%) classified as malignant with white light and NBI, respectively, were found to be malignant on pathological examination.

**Table 4:** Scoring of all lesions into three categories (benign, dubious, or malignant)

	White light		NBI	
Intraoperative assessment	Number of lesions	Malignant on pathological examination (%)	Number of lesions	Malignant on pathological examination (%)
Benign (VAS 1–4)	93	5 (5)	88	2 (2)
Dubious (VAS 5–6)	19	7 (37)	2	0 (0)
Malignant (VAS 7–10)	57	38 (67)	79	48 (61)

The first column for both modalities describes the number of lesions in each category, while the second column for each modality describes the number and percentage of this category that was found to be malignant on pathological examination

*VAS* visual analog scale, *NBI* narrow-band imaging

## Discussion

This clinical feasibility study clearly demonstrates NBI to be superior to white-light imaging, NIR-ICG, and SDCE for the detection of colorectal PM. These results encourage the use of NBI for enhanced visualization of PM during inspection of the peritoneum at the time of primary tumor resection and realization of a complete CRS prior to HIPEC treatment. In 28 patients with colorectal PM, NBI improved sensitivity for the detection of PM from 80.0% with white-light imaging to 96.0% (*p* = 0.008). The use of NBI alone did not significantly impair specificity (74.8% vs. 73.1%; *p* = 0.804), although the combined use of NBI and white light did. Using NBI, lesions were identified that would have been missed with white-light imaging. Thereby, this technique provided guidance to categorize lesions that were dubiously positive with white light.

NBI is available on most laparoscopic systems and is a practical method not requiring extra costs or significant additional time. This method has already been widely studied for its use during colonoscopy^26^ and the detection of other primary tumors,^18^^,^^19^^,^^27^ but its use for the detection of PM has been reported less frequently. Although NBI enhanced sensitivity for the detection of PM in 26 gastric cancer patients, from 48 to 91%,^18^ its additional value was not shown in 20 patients with gastrointestinal and gynecological malignancies.^28^ The latter study did not provide clear alignments for classification of lesions and did not assess interrater variability, making these results subject to individual variation in interpretation. More importantly, only one patient in this heterogeneous cohort had colorectal PM, severely hampering the conclusions of this study. Furthermore, a recent study including 124 patients with gynecological cancer did not find NBI to be superior to white-light imaging for the detection of PM.^29^ However, it is questionable whether the conclusions on PM of mucinous ovarian cancers can be extrapolated to PM of colorectal origin. The present prospective evaluation of three advanced imaging techniques in a computer-randomized order does suggest a role for NBI in the detection of colorectal PM. Notably, combining white light with NBI resulted in a higher false positive rate, potentially leading to unnecessary resections. In addition, the slightly higher VAS assigned to both benign and malignant lesions using NBI suggests that not only pathologically confirmed malignant lesions but also benign lesions are more likely to be scored as malignant. Nevertheless, the oncologic benefits of a better tumor resection are expected to outweigh the potential risks of a more extensive surgical resection.

In the present study, SDCE with indigo carmine blue was not considered suitable for application onto the whole abdominal cavity and intraperitoneal organs, even impairing visualization of intra-abdominal organs, while assessment of the complete peritoneal lining is crucial to achieving a complete cytoreduction. In particular, abdominal regions less easily approachable by laparoscopy cannot be properly evaluated using this technique. To date, only one case report has described the intraperitoneal use of SDCE, and found this method to be of potential benefit for the identification of endometriotic lesions.^30^ Considering the lack of visualization of all potentially affected surfaces, which may impair oncologic outcome, its application in terms of cancer detection will probably be limited to characterization of previously identified and localized lesions, such as early gastric cancer lesions,^31^ and to its use during colonoscopy.^23^

No fluorescence was detected in the present study using NIR-ICG. The timing of ICG administration may be an explanation for the lack of fluorescence in the present study. The optimal timing of ICG administration is highly debated, ranging from intraoperative administration^20^ to 1–24 h preoperatively.^21^^,^^22^^,^^32^ These contradictory recommendations are in line with the conflicting results on NIR-ICG for the detection of PM. Although two small studies (*n* = 10) reported relatively low sensitivities of 65% and 76%,^22^^,^^32^ the ex vivo use of tumor-to-background ratios after intraoperative ICG administration resulted in a relatively high sensitivity (88%). Unfortunately, the value of NIR-ICG was limited for the detection of mucinous tumors and in areas with high physiological ICG accumulation, such as the liver, as well as areas with a high peritoneal tumor load.^20^ In our institution, ICG is routinely administrated intraoperatively during laparoscopic segment resections to assess anastomotic vascular sufficiency. However, in patients with PM, no peritoneal deposits have been visualized using this technique, which may be partly explained by high background fluorescence. Therefore, the time interval in the present study was based on the enhanced permeability and retention principles.^32^^,^^33^ Currently, fluorescence with antibody-coupled ICG is an emerging technique that shows promise. Both the use of fluorescent monoclonal carcinoembryonic antigen antibodies^34^ and ICG-coupled antibodies targeting vascular endothelial growth factor^35^ are promising methods, revealing tumor tissue that has been missed with white light. A comparative study evaluating promising dyes should determine the optimal target for this imaging modality. Until now, these techniques are not widely available in clinical practice and their implementation will require substantial investment in time and money.

The main potential clinical implication of NBI is the detection of PM during evaluation of the peritoneum at the time of laparoscopic primary tumor resection. Early detection of PM and subsequent treatment is crucial for the successful treatment of patients with PM. In particular, patients with T4 tumors deserve a dedicated inspection since T4 CRC is a major risk factor for PM, with up to 20% of these patients presenting with synchronous PM.^36^–^38^ NBI could help detect peritoneal disease in an early stage, resulting in early referral to specialized HIPEC centers and, consequently, improved outcome. A second implication is determination of the feasibility of a complete CRS at the time of diagnostic laparoscopy. Currently, 25–44% of CRC patients considered eligible for CRS and HIPEC based on preoperative imaging undergo unnecessary explorative procedures.^39^^,^^40^ This number was shown to be reduced by 25–35% with prior laparoscopic assessment in colorectal, ovarian, and gastric cancer patients.^40^–^45^ However, it should be noted that the Peritoneal Cancer Index (PCI) is often underestimated during diagnostic laparoscopy,^46^ and a relatively disappointing PPV of 83% for prediction of complete cytoreduction has been reported.^47^ A third implication is the optimization of a radical resection at the time of CRS, although the NBI system is less practical for use during open surgery. Notably, improved detection of PM may reveal higher PCI scores. The mean size of the lesions missed with white light was > 5 mm, implying that proper visualization of these lesions would impact PCI scores and that patients who would have been treated with curative intent based on white-light imaging might have received palliative treatment based on advanced imaging. Therefore, it is of utmost importance to investigate whether the visual advantages indeed translate into therapeutic benefit for patients in terms of tumor staging and oncologic outcomes, warranting randomization between imaging techniques and a long-term follow-up.

This prospective clinical study is the first to demonstrate that NBI improves the detection of colorectal PM. However, some limitations should be taken into account. First, we could not compensate for a potential learning curve regarding assessment of the lesions. This problem was encountered by providing photographs of all lesions, clear scoring guidelines, and evaluation of all lesions by two independent surgeons. Second, we did not use ex vivo evaluation systems, such as tumor-to-background ratios, that could have resulted in higher sensitivities. Nevertheless, real-life assessment of imaging techniques provides a better indication for their use in clinical practice. The risk of taking non-representative biopsies should also be noted. This issue was addressed by careful documentation of the locations of scored and biopsied lesions and by recording all surgical procedures.

## Conclusion

NBI is a safe and practical option that could help realize the early detection of PM during evaluation of the peritoneum at the time of primary tumor resection, and subsequently improve CRS. The influence of this modality on clinical decision making and oncologic outcomes should be examined in prospective studies comparing NBI and white-light imaging. Future studies assessing promising advanced imaging techniques, such as NBI and molecular fluorescence-guided techniques, should provide the information necessary to determine the place of advanced imaging techniques in surgery.

## Electronic supplementary material

Below is the link to the electronic supplementary material.

Supplementary material 1 (DOCX 15 kb)
