# Supplementary material for: Narrow-Band Imaging Improves Detection of Colorectal Peritoneal Metastases: A Clinical Study Comparing Advanced Imaging Techniques
Source: Ann Surg Oncol. 2018 Nov 12;26(1):156–64. doi: 10.1245/s10434-018-7005-5 (PMC6338718; doi:10.1245/s10434-018-7005-5)
Supplement: Supplementary file 1 — Supplementary material 1 (DOCX 15 kb) [file 10434_2018_7005_MOESM1_ESM.docx]

# Supplementary Methods

### *Study Design and Patients*

Patients with colorectal PM scheduled for CRS and HIPEC were prospectively enrolled in this clinical feasibility study. Primary outcomes were sensitivity and specificity for detection of colorectal PM. Secondary outcomes were PPV, NPV, LR+, and LR−. In addition, safety of the imaging techniques was assessed. The study was approved by the local Investigational Review Board of the Amsterdam UMC, Vrije Universiteit Amsterdam (METC VUmc 2015.257), and was registered in the Dutch Trial Register before the start of the study (NTR5820, http://www.trialregister.nl/trialreg/admin/rctview.asp?TC=5820). The study procedures were undertaken with the understanding and appropriate informed consent of all patients.

Patients were excluded if they were younger than 18 years, legally or mentally incapable or unable to give informed consent, they had an American Society of Anesthesiologists (ASA) score more than three, contraindications for laparoscopy such as prior major abdominal surgery, PM of non-colorectal or LAMN origin, or absence of PM. Patients were also excluded in case of a contraindication for the use of indocyanine green or indigo carmine blue: hypersensitivity, use of interacting medication, cardiac disease, chronic kidney failure (eGFR <55) or chronic liver failure (ALAT, ASAT, AFP, or GGT more than two times the maximal normal value).

Demographic data, primary tumor information, information on prior treatment and the CRS and HIPEC procedure were prospectively collected from the patient records. Additionally, information on Prior Surgical Scores (PSS),^12^ PCI,^12^ and resection scores^13^ was retrieved.

### *Surgery*

All included patients underwent a diagnostic laparoscopy prior to CRS and HIPEC during which NBI, NIR-ICG, and SDCE were investigated and compared with two-dimensional, high-definition white-light imaging. For the diagnostic laparoscopy, a deflectable videoscope (CLV-180 EVIS EXERA II platform, Olympus, Center Valley, PA, USA) was introduced through a 12 mm trocar. Two additional 5 mm trocars were introduced to facilitate maximal exposure of the abdominal cavity. Subsequently, a specialized gastrointestinal surgeon inspected the peritoneum systematically according to the PCI. If the extent of peritoneal disease was deemed resectable, laparoscopy was followed by conversion to an open laparotomy and CRS and HIPEC. CRS consisted of removal of all visible tumor, stripping of the affected parietal peritoneum, including the peritoneum of the liver and diaphragm, resection of the omentum and, when applicable, removal of adnexa and multi-organ resections.^48^ In case of a complete cytoreduction,^13^ a HIPEC procedure was carried out during which mitomycin C was administered intraperitoneally with a target temperature of 41°C for 90 min. All intra- and postoperative adverse events were recorded.

### *Imaging modalities*

After examining all 13 abdominal regions by white-light imaging during laparoscopy, the peritoneum was inspected with NBI, NIR-ICG, and SDCE in a computer-randomized order generated by the online available Sealed Envelope Ltd program (London, UK). Imaging techniques considered unsuitable for clinical practice, based on difficulty of use, length of the procedure, or lack of fluorescence, were excluded from the study after agreement between the leading investigator and the operating surgeons.

Narrow-Band Imaging Narrow-band imaging was investigated using the laparoscopic CLV-180 EVIS EXERA II platform with an ENDOEYE EXERA II deflectable videoscope. Lesions were scored as malignant if they showed signs of vessel dilatation, vessel tortuousness, vessel heterogeneity, or brown spots.^18,19^

Near-Infrared Imaging with Indocyanine Green

Indocyanine green (ICG-PULSION®, PULSION Medical Systems AG, Munich, Germany) was diluted in sterile water (0.25–0.50 mg/kg bodyweight) and injected intravenously 3–12 h prior to surgery. The Olympus VISERA Pro (Olympus, Center Valley, PA, USA) laparoscopic platform was used with a modified near-infrared light filter (800 nm) on the videoscope that filtered near-infrared reflection in the range of 700–900 nm. Lesions were scored as malignant if they appeared fluorescent using NIR-ICG.

*Spray-Dye Chromoendoscopy*

Indigo carmine blue was sprayed onto the peritoneum using a sterile dye-spray catheter. After staining of the peritoneum, the indigo carmine solution was rinsed with 0.9% sodium chloride and aspirated. Lesions were considered malignant if SDCE revealed a lesion by contrast enhancement.

### *Sample Size*

The sample size was based on the estimated number of malignant peritoneal lesions necessary to achieve a power of 80% to detect an increase in sensitivity from 75% with white-light imaging to 95% with one of the advanced imaging modalities. Power analysis revealed a number of 57 pathologically confirmed malignant lesions required to detect a difference with a two-sided McNemar’s test at a significance level of 0.05. Based on an estimated 6–8 lesions per patient and a positive rate of 33%, a sample size of 25–30 patients is required.
